# Unusual cystic sebaceous neoplasm prompts cascade testing

**DOI:** 10.1016/j.jdcr.2024.02.026

**Published:** 2024-03-07

**Authors:** Ryan A. Hotchkiss, Felix Yang, Mary Beth Gadarowski, Michael P. Orejudos, Carolyn A. Hardin Robinson

**Affiliations:** a Uniformed Services University of the Health Sciences, School of Medicine, Bethesda, Maryland; b Department of Dermatology, San Antonio Uniformed Services Health Education Consortium, San Antonio, Texas; c Department of Pathology, San Antonio Uniformed Services Health Education Consortium, San Antonio, Texas

**Keywords:** cancer screening, CHEK2 gene, cystic sebaceous neoplasm, genetic testing, Muir-Torre syndrome

## Introduction

The differential diagnosis of flesh-colored papules or nodules is broad, including a number of benign and malignant neoplasms. Along the head and neck, they may represent common entities such as sebaceous hyperplasia or a number of adnexal tumors. It remains important to maintain an appropriate level of concern for more malignant processes such as basal cell carcinoma, squamous cell carcinoma, or sebaceous neoplasm (SN) that represent cutaneous manifestations of Muir-Torre syndrome (MTS).^1^

MTS involves at least 1 SN and 1 visceral malignancy. Other factors such as family history of malignancy, presence of multiple keratoacanthomas, and tumor location have been incorporated into a clinical tool to assess the pre-test probability for MTS,^1^ and thus screening for MTS and other DNA mismatch repair cancer syndromes requires attentive synthesis of clinical presentation and history.

We present the case of a 54 year-old-male with a painful, subcutaneous nodule on the lower back with oily drainage and cyst wall observed during surgical excision. Histopathological assessment favored a SN, and immunohistochemical studies demonstrated loss of mismatch repair proteins, prompting a high suspicion for MTS. Subsequent genetic evaluation instead revealed a pathogenic checkpoint kinase 2 (CHEK2) mutation which triggered cascade testing.

## Case report

A 54-year-old Fitzpatrick II male with a history of stage 1A melanoma presented to dermatology for a 3 × 2 cm painful and well-circumscribed subcutaneous nodule on his lower back. His only notable skin cancer history was a prior melanoma status post local excision in 2014. His family history was significant for a mother and maternal aunt who passed away in their 40s from metastatic cancers of unknown primaries.

**Fig 1:**
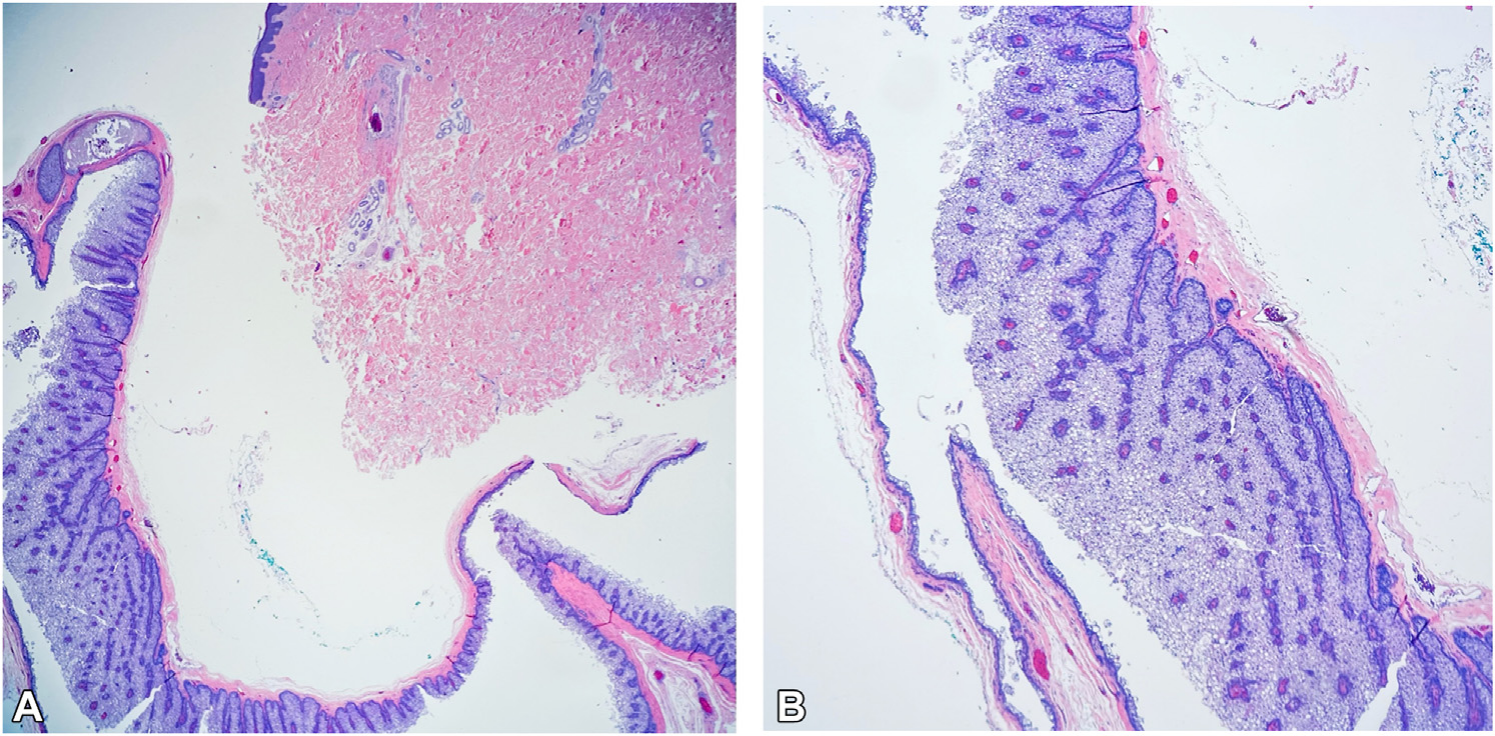
**A,** Cystic sebaceous neoplasm hematoxylin & eosin. Low power view of predominantly cystic architecture; 10× magnification. **B,** Cystic sebaceous neoplasm hematoxylin & eosin. Predominantly cystic architecture, cont; 40× magnification, zoomed in.

**Fig 2:**
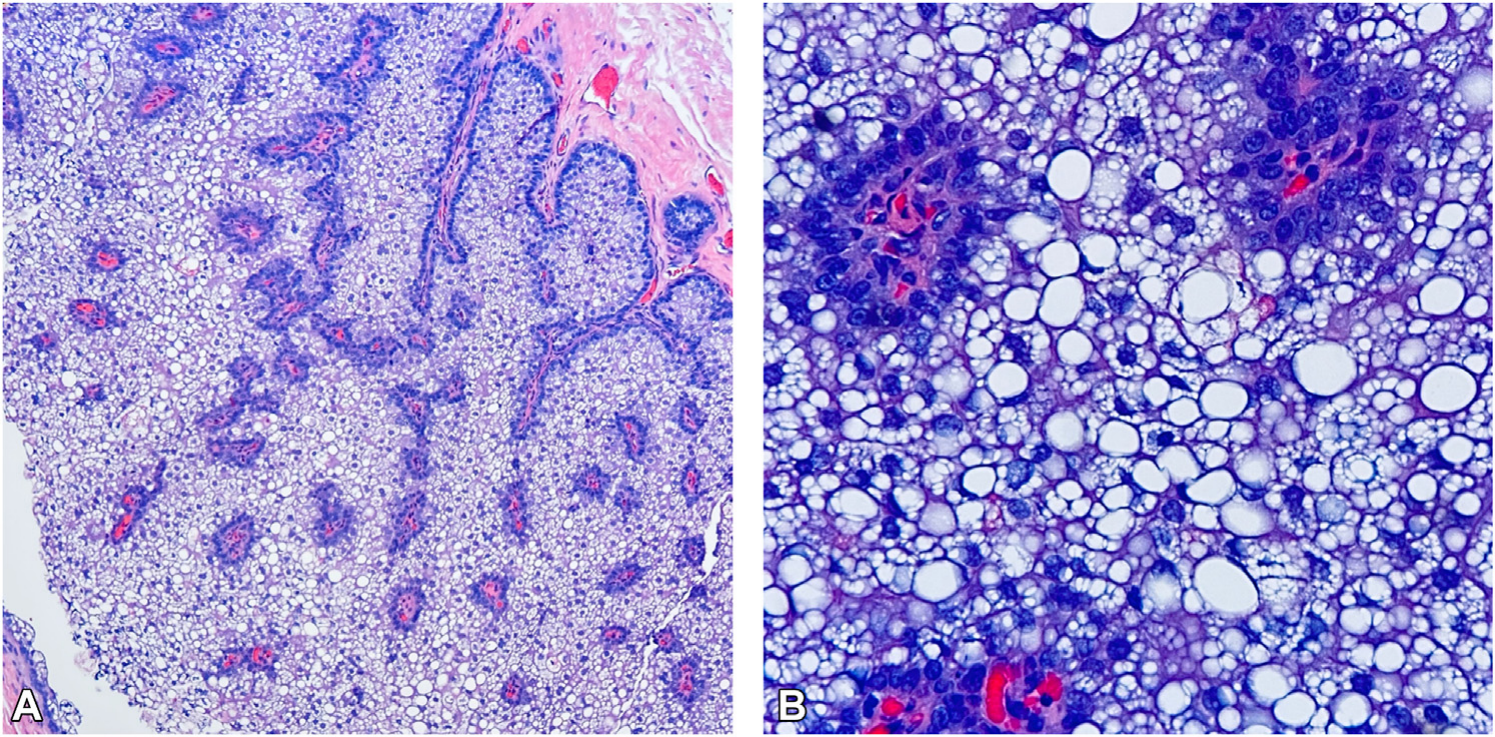
**A,** Cystic sebaceous neoplasm hematoxylin & eosin. Cystic lining composed of sebaceous cells with peripheral germinative layer of small basaloid cells, with mature sebaceous cells centrally and transition forms in between; 100× magnification. **B,** Cystic sebaceous neoplasm hematoxylin & eosin. Higher power view demonstrating mature cells outnumber the darker germinative cells; 400× magnification.

Upon initial surgical excision, the lesion demonstrated a cyst wall and oily drainage. Histological examination was most consistent with a cystic SN. These unusual features raised significant concern for MTS [Figs 1 and 2, *A* and *B*] and warranted subsequent immunohistochemical evaluation.^2^

**Fig 3:**
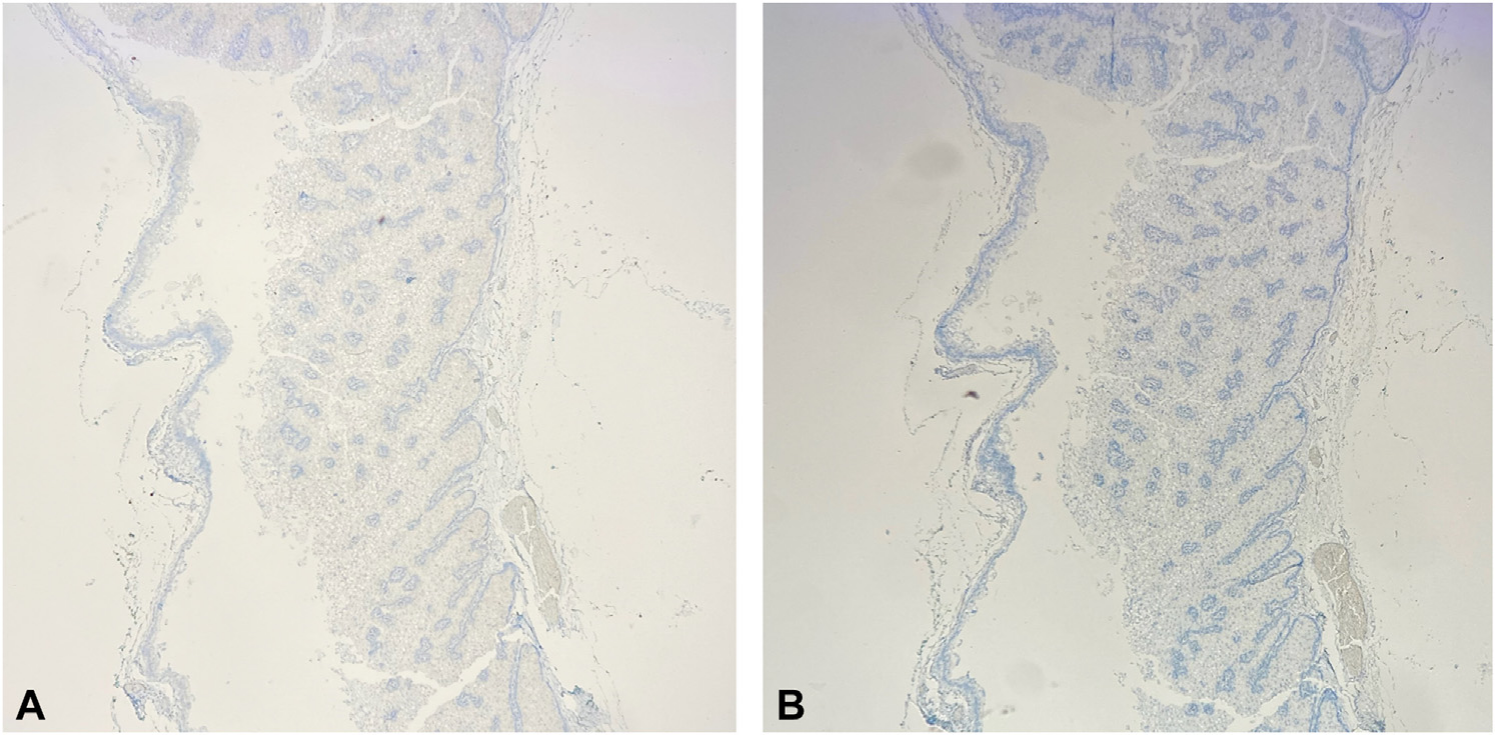
**A,** Cystic sebaceous neoplasm immunohistochemical stain at 40× magnification for postmeiotic segregation increased 2 demonstrating loss of expression. **B,** immunohistochemical stain at 40× magnification for mutL homolog 1 demonstrating loss of expression.

A reexcision at a later date produced clear surgical margins without evidence of malignancy. The Mayo MTS risk calculator produced a score of 1 for the patient (younger than 60 years old at first diagnosis of SN),^1^ and subsequent immunohistochemical testing per diagnosis and management recommendations revealed loss of nuclear expression of mismatch repair proteins mutL homolog 1 and postmeiotic segregation increased 2 [Fig 3, *A* and *B*].^2^ Broad testing via the Invitae Lynch Syndrome and Multi-Cancer panels was performed because of the nebulous family history of metastatic cancer of unknown primary which revealed a pathogenic CHEK2 heterozygosity, associated with breast, prostate, and colorectal cancers^3^^,^^4^ prompting cascade testing for first-degree relatives.

## Discussion

The factors that prompted further histologic and genetic evaluation of this cystic SN were the atypical location on the lower back, associated pain, oily drainage and cyst wall observed during surgical excision, and the family history of metastatic disease of unknown primary. The discovery of a cystic SN on the back necessitated further workup for MTS.

MTS is a variant of Lynch syndrome and commonly presents with mutations in the mutL homolog 1, mutL homolog 2, mutL homolog 6, and postmeiotic segregation increased 2 mismatch repair genes,^5^ which can predispose patients to visceral malignancies, especially colonic carcinomas.^5^ Genetic testing was negative for Lynch-associated genes, but unexpectedly revealed a pathogenic heterozygous CHEK2 mutation, a gene associated with increased risk of breast, prostate, kidney, thyroid, and colorectal cancers.^6^

CHEK2 is an oncogene that codes for the checkpoint kinase 2 protein involved in DNA double-strand break repair.^6^ It is inherited in an autosomal dominant pattern and has the highest prevalence among Caucasian individuals of European descent. There are a variety of pathogenic CHEK2 mutations, with the c.1100del mutation identified in our patient being the most widely studied.^6^ Although there are no specific prostate or colon cancer screening guidelines for CHEK2, National Comprehensive Cancer Network guidelines support shared decision-making for prostate cancer screening at the age of 40 or earlier depending on family history for individuals with germline mutations associated with colon and prostate cancer.^7^ Our patient has had yearly prostate specific antigen screening for over 10 years (given testosterone supplementation for hypogonadism) and he is to follow with urology to evaluate his risk for prostate cancer. At the time of writing, this patient is due for colon cancer screening.

With regards to breast cancer, CHEK2 is considered a moderate-high risk gene and has been shown to be associated with an increased risk of male breast cancer in the Netherlands. Although there are no CHEK2 specific guidelines for breast cancer screening in male carriers, monthly self-breast exams with yearly clinical exams are recommended for men with high risk genetic predisposition to breast cancer.^8^^,^^9^

It is not yet established how CHEK2 contributes to the development of SNs, if at all, and further research may be focused toward possibly additive effects of deficient mismatch and double-strand break repair. An extraocular cystic SN by itself should raise concern for MTS, and while the patient’s clinical history and histological findings were initially suggestive of MTS, genetic testing led down an entirely different route which has now resulted in refined screening for him and cascade testing in his first-degree relatives. With timely suspicion of cancer syndromes, dermatologists can set into motion genetic counseling and screening to mitigate the impact of pathologic mutations on a patient’s life.

## Conflicts of interest

None disclosed.
